# Space-Valence Priming with Subliminal and Supraliminal Words

**DOI:** 10.3389/fpsyg.2013.00081

**Published:** 2013-02-22

**Authors:** Ulrich Ansorge, Shah Khalid, Peter König

**Affiliations:** ^1^Department of Psychology, University of ViennaVienna, Austria; ^2^Institute of Cognitive Science, University of OsnabrückOsnabrück, Germany; ^3^Department of Neurophysiology and Pathophysiology, University Medical Center Hamburg-EppendorfHamburg, Germany

**Keywords:** priming, embodied cognition, unconscious processing, emotions, metaphors

## Abstract

To date it is unclear whether (1) awareness-independent non-evaluative semantic processes influence affective semantics and whether (2) awareness-independent affective semantics influence non-evaluative semantic processing. In the current study, we investigated these questions with the help of subliminal (masked) primes and visible targets in a space-valence across-category congruence effect. In line with (1), we found that subliminal space prime words influenced valence classification of supraliminal target words (Experiment 1): classifications were faster with a congruent prime (e.g., the prime “up” before the target “happy”) than with an incongruent prime (e.g., the prime “up” before the target “sad”). In contrast to (2), no influence of subliminal valence primes on the classification of supraliminal space targets into up- and down-words was found (Experiment 2). Control conditions showed that standard masked response priming effects were found with both subliminal prime types, and that an across-category congruence effect was also found with supraliminal valence primes and spatial target words. The final Experiment 3 confirmed that the across-category congruence effect indeed reflected priming of target categorization of a relevant meaning category. Together, the data jointly confirmed prediction (1) that awareness-independent non-evaluative semantic priming influences valence judgments.

## Introduction

Human evaluation or appraisal processes concern the assignment of positive and negative valence to things and events (Arnold, 1960; Lazarus, 1968; Scherer et al., 2001; Storbeck and Clore, 2007). Such appraisal processes are very important for human ontogenetic and phylogenetic fitness (Arnold, 1960; LeDoux, 1998). The appraisal of things and events is made with respect to an organism’s vital goals. Appraisal can thus guide the selection of crucial stimulus information and goal-directed actions for utility maximization because utility is based on the achievement of merited or positively evaluated outcomes and the avoidance of negative consequences (Posner and Dehaene, 1994; Pessoa et al., 2002). In fact, humans carry out evaluations so routinely and automatically (Kissler et al., 2007) that the corresponding appraisal processes can be triggered by irrelevant words, and even by stimuli remaining outside of the awareness of humans, such as subliminal or unconscious words or tokens (Naccache et al., 2005; Galliard et al., 2006; Klauer et al., 2007; Pessiglione et al., 2007). These studies suggest a fast evaluation system that operates independent of conscious perception.

Despite their enormous significance, however, to date we do not fully understand how such automatic evaluations relate to other non-evaluative dimensions of semantic processes. Here, we refer to semantic processes as the processes concerned with the assignment of meaning in general (Osgood et al., 1957; Meyer and Schvanefeldt, 1976; Kintsch and van Dijk, 1978; Rey, 2003). According to some theories, valence would be one integral dimension of all semantic meaning (Osgood et al., 1957). Even if this is true, however, it remains to be specified whether evaluative meaning is as quickly available as non-evaluative meaning or whether non-evaluative meaning precedes evaluative meaning. One possibility is that evaluations are special and carried out so quickly (Zajonc, 1980; LeDoux, 1998) as to precede and ground visual recognition and subsequent processing of non-evaluative forms of meaning. In line with this, for example, visual stimuli with a conditioned valence can elicit valence-dependent changes of visually evoked potentials of the human EEG with a latency of less than 100 ms (Stolarova et al., 2006). Related to this, Naccache et al. (2005), for example, demonstrated that visually masked subliminal words elicited valence-dependent activation of the human amygdala, a brain structure involved in emotional processing (LeDoux, 1998).

On the other hand, some emotion theories argued that visual recognition and specific non-evaluative semantic inferences have to precede appraisal and evaluative processes (Storbeck and Clore, 2007; Nummenmaa et al., 2010). Specifically, according to the embodied cognition view, word meaning is always grounded in more basic representations, such as sensory or sensorimotor representations (Barsalou, 1999, 2008; Glenberg and Kaschak, 2002; Zwaan et al., 2002). Thus, valence could be accessible only after some other forms of non-evaluative sensory meaning have been successfully extracted from a word. In line with this, participants in a study of Nummenmaa et al. (2010), for example, were able to saccade toward one of two scenes based on a scene’s semantic content before they were able to saccade to a scene based on a scene’s valence or affective content. Likewise, Niedenthal (2007) argued that emotions could in general be grounded in preceding basic sensory, perceptual, and sensorimotor representations of postures and facial expressions. These divergent claims are the point of departure for the present research.

We address the directed interaction of valence evaluation, semantic processing, and conscious perception. As follows from the above, a key issue is how quickly the valence of a word becomes available. Is valence accessible before non-evaluative forms of meaning, especially sensory-related semantic meaning? Or is the sensory-related and non-evaluative meaning of a word accessible before its valence? For our test, we used an across-category priming effect of sensory representations on valence recognition: the influence of spatial verticality on valence classification where spatially high corresponds to positive valence, and spatially low to negative valence. Of course, spatial location is but one of the sensory representations that nurtures the embodied representation of emotions. For instance, facial expressions are also among the powerful sensory representations that link up to emotions (Niedenthal et al., 2009). However, the association between valence and space is strong, robust, and already reflected in the very meaning of some words (Lakoff and Johnson, 1980; Melara and O’Brien, 1987). For example, the spatial word “*high*” can be used almost synonymously to denote an affective state of “*euphoria*” and the spatial word “*low*” for an affective state of “*depression*.” In their seminal experiments, Meier and Robinson (2004) demonstrated this modulating influence of spatial verticality on valence in a *space-valence congruence effect*: their participants were faster when discriminating the valence of positive words above screen center and of negative words below screen center. This advantage in space-valence congruent conditions was found in comparison to incongruent conditions, in which the negative words were presented above screen center and the positive words below screen center (for related results, see Crawford et al., 2006; Weger et al., 2007; Horstmann, 2010; Horstmann and Ansorge, 2011; Santiago et al., 2012; Ansorge and Bohner, in press; Gozli et al., 2012).

So far, this space-valence congruence effect has not been tested with subliminal words. This, however, is important. If we can demonstrate an across-category interaction with subliminal words (Forster, 1998; Ansorge et al., 2010) then the space-affect congruence interaction during lexical access reflects a form of early automatic impact of either quick semantic processes on evaluations or of quick evaluative appraisal on semantic processing. For example, Lamme (2003) and Lamme and Roelfsema (2000) estimated that unaware processing occurs during the first 100 ms post-stimulus. The exact duration might be slightly longer (Mulckhuyse and Theeuwes, 2010) but authors share the view that awareness-independent processing occurs early, during the feed-forward phase of stimulus processing, whereas awareness-dependent processing takes time and occurs later because it depends on feedback from processes higher up the hierarchy (Lamme, 2003). This implies limitations on subliminal processing. For example, subliminal processing could sometimes be conditional on top-down task sets. To be precise, subliminal processing could be “conditionally automatic,” meaning that it depends on the task-relevance of the unaware stimulus (e.g., of a semantic dimension of a subliminal word; Bargh, 1992). Yet, importantly, this also means that a subliminal word does not elicit a fitting intention for its own processing in and by itself (Forster, 1998; Kinoshita et al., 2011). On the contrary, before the processing of subliminal stimuli can take place the prior set-up of a top-down or goal-directed intention would have to be firmly established (Klinger et al., 2000; Kunde et al., 2003). Only once such an intention, goal, or task set has been firmly established, a stimulus meaning of which a person remains unaware would be able to elicit a stimulus processing in the feed-forward processing phase (e.g., Norris and Kinoshita, 2008; Ansorge et al., 2010, 2012). Note that this general assumption also holds true for the processing of a subliminal word’s valence meaning (Klinger et al., 2000; De Houwer et al., 2002; Klauer and Musch, 2002; Eckstein and Perrig, 2007; see also Spruyt et al., 2009; Spruyt et al., 2012).

Here, to test the predictions, and to understand whether sensory semantics quickly and (conditionally) automatically affected evaluations during the feed-forward phase and/or whether this is the other way round, we used (1) subliminal spatial words as primes and valence words as targets (Experiments 1 and 3), or we used (2) subliminal valence words as primes and spatial words as targets (Experiments 2 and 3). If valence meaning is based on (connotative) spatial representations, we expected a congruence effect of space primes on target evaluations (in Experiment 1). In the congruent condition, a valence judgment should be facilitated. For example, the prime word “up” should facilitate classifying the emotional target “happy” as positive. Facilitation was expected compared to the incongruent condition, for example, when the prime word “down” was presented prior to the emotional target “happy.” If the expected congruence effect reflected a quick, mandatory (or conditionally automatic) process, this space-valence congruence effect should be found with subliminal space primes, too (Ansorge et al., 2010). In addition, if word valence is as quickly available as a word’s spatial meaning, we should find a congruence effect in the reversed situation (in Experiment 2) in which subliminal valence primes were presented before spatial targets.

When related predictions have been tested concerning the symmetry versus asymmetry of the space-valence congruence effect in the original paradigm of Meier and Robinson (2004), researchers observed congruence effects based on irrelevant spatial positions during word evaluation but no influence of irrelevant word valence on the discrimination of word locations (Santiago et al., 2012; but see Experiment 3 of Meier and Robinson, 2004). However, Santiago et al. achieved their results with clearly visible words and locations, leaving it open whether a similar asymmetry of space-valence congruence originates during the feed-forward phase of processing. In addition, when more attention was shifted to the irrelevant valence of the clearly visible words, the space-valence congruence effect was reestablished even during spatial discrimination (Experiment 6 of Santiago et al., 2012).

In our study, as control conditions, we therefore used (1) blocks with clearly visible, so-called “supraliminal” primes and targets from different categories (and from the same categories) and (2) trials with subliminal primes and targets from the same category (e.g., a valence prime before a valence target) instead of primes and targets from different categories in both Experiments 1 and 2. In the control conditions, several hypotheses predict a congruence effect. With the supraliminal primes, a congruence effect could be based on strategic rather than (only) quick, obligatory (or conditionally automatic) processing of the visible prime because the longer perceptual trace of the visible primes affords also more attentional dwelling on visible than masked primes: the clearly visible prime will be seen and can elicit its strategic processing in and by itself, so that processing of the priming word would no longer be dependent on a preceding fitting task set (Cheesman and Merikle, 1985; Forster, 1998). For example, in the study of Cheesman and Merikle, participants strategically used the predictive power of the categories of the visible prime words of male and female gender names for the target word categories of female and male gender names, respectively. This was evident in the efficient preparation of the most likely target word responses. This strategic effect was found when the prime words were visible but not when the prime words were invisible (see also, e.g., Kinoshita et al., 2011). It is possible that this boosting influence of strategies on priming is due to a fundamental difference between feed-forward processing and recurrent processing: due to the participants’ awareness of the visible prime, this prime word might be broadcasted to different processing modules throughout the mental sphere and could thus be used for multiple new purposes, including the strategic assessment of the fit of its meaning relative to that of the target (Baars, 1988; Dehaene and Naccache, 2001). Therefore, the standard space-valence congruence effect should be found in the supraliminal conditions and a difference might be expected between supraliminal and subliminal conditions: if one of the prime word meaning dimensions (spatial or evaluative) contributing to the space-valence association is not also quickly and automatically processed (and thus could not be processed during the early feed-forward processing phase), then we expect that this dimension should become effective in the supraliminal but not in the subliminal conditions.

Also, in the second type of control conditions, with the subliminal primes of the same category, a congruence effect of the masked primes is predicted based on the prime’s potential of priming a motor response (Neumann, 1990; Kunde et al., 2003; Ansorge and Neumann, 2005). Say, one needs to press the left button for a positive target word and a right button for a negative target. A positive prime before a positive target would then indicate giving the same response but a negative prime before a positive target would cause response conflict. Because this response-activation effect has already been demonstrated for subliminal prime words (Damian, 2001) – a principle termed “action triggering” (Kunde et al., 2003) – at least in the within-category priming conditions a congruence effect should be found with the subliminal primes. In the subliminal conditions with primes and targets from the same category, we can expect a congruence effect, thus, making sure that our methods are sensitive enough to reveal a subliminal priming effect even if this priming effect happens to fail in the subliminal across-category (space-valence or valence-space) priming conditions.

To conclude, two origins of the congruence effects are conceivable in the present context: semantic priming (or category priming) – that is, decision priming in favor of one meaning or one category (Collins and Loftus, 1975; Plaut and Booth, 2000); and response priming (Klinger et al., 2000; Kunde et al., 2003). Semantic or category priming (Greenwald et al., 1996, 2003; Naccache and Dehaene, 2001; Kiefer, 2002; Norris and Kinoshita, 2008; Martens et al., 2011) was expected in the across-category priming conditions. Response priming (and maybe semantic or category priming) was expected in the within-category priming conditions only (Damian, 2001; Kunde et al., 2003).

Prior research has shown that the different origins of the congruence effect can be discriminated on the basis of their development over time [i.e., over the reaction time (RT) distribution]. According to Kinoshita and Hunt (2008), the priming of a decision for or against one category of semantically defined targets would be reflected in a temporally stable congruence effect. This congruence effect would be present in about similar strength across all of the RT distribution, from fast to slow responses. Thus, a congruence effect based on category priming should be found in fairly equal amounts for all RTs, from the fastest to the slowest.

By contrast, according to Kinoshita and Hunt (2008), a congruence effect based on response priming should decrease across time (i.e., across the RT distribution) in a manner different from a category priming effect. A response priming effect should be stronger among the faster responses and it should decrease among the slower responses. To understand the origin of the expected congruence effects in the current study, we therefore tested the congruence effects as a function of the RT.

## Experiment 1

In Experiment 1, our participants had to categorize the clearly visible valence targets as either positive (e.g., the target “*joyful*”) or negative (e.g., the target “*sad*”) in a 2 (within/across-category) × 2 (congruent/incongruent) × 2 (visible/masked) design. They had to press one of two keys (left key versus right key) to classify each target. Prior to every target, a prime word was shown. In half of the trials of the across-category priming condition, the prime was a spatial up-word (e.g., the word “*above*”), and in the other half it was a down-word (e.g., the word “*below*”). Together, these were the spatial priming conditions. In the within-category priming condition, in half of the trials the prime was a word of positive valence (e.g., the prime “*happy*”), and in the remaining trials it was of negative valence (e.g., the word “*frustrated*”). Together these were the valence priming conditions.

In the congruent conditions, primes and targets had associated meanings. For example, in the within-category condition a positive prime could have been presented before a positive target, while in the across-category condition an up-prime could have been presented before a positive target. In the incongruent conditions, primes and targets had less associated meanings. For example, in the within-category condition a negative prime could have been presented before a positive target, while in an across-category condition an up-prime could have been shown before a negative target.

In one block, the primes were presented as clearly visible words. In another block, the primes were presented subliminally, here: masked. In this context, masking denotes an experimental procedure where a visual stimulus replaces a preceding word so as to suppress the word’s visibility (Marcel, 1983). To ensure that the masked primes were truly subliminal, prime visibility was individually tested, and participants that were suspiciously good during the discrimination of the masked primes were excluded^1^.

On the basis of prior research (Meier and Robinson, 2004), we expected an across-category, space-valence congruence effect with the visible primes. Responses should be faster in congruent than incongruent conditions. Critically, if sensory (here spatial) meaning can be extracted swiftly and (conditionally) automatically from the priming words, we might find a space-valence congruence effect in the visible *and* in the subliminal priming conditions – that is, regardless of awareness.

### Method

#### Participants

The participants of Experiment 1 and of the other experiments had normal or corrected-to-normal vision, were mostly university students and given course credit for participating. Two participants had to be excluded because of too high a number of correct prime-target judgments in the masked condition (see text footnote 1), and two further participants had to be excluded because of chance performance in the prime-target judgments of the unmasked condition, indicating that they were unable or unwilling to discriminate the unmasked prime-target relations. The remaining 40 participants (31 female, *M*_age_ = 22.0 years, age range: 18–28 years) were analyzed.

#### Apparatus, stimuli, and procedure

Prime and target stimuli were German words denoting emotional adjectives or prime stimuli were spatial words denoting directions or positions on the vertical axis. We used the following prime and targets that were all high frequency words because there were more than 60 instances in 1 million words (Jescheniak and Levelt, 1994), with frequencies word counts in parentheses according to the Wortschatz Lexikon of the University of Leipzig, http://dict.uni-leipzig.de/, and frequencies calculated relative to 400,000 entries, contained in the Wortschatz Lexikon on the date of retrieval, December 5, 2012). As positive valence words, we used: “*lustig*” (*jolly*; 2,521), “*glücklich*” (*lucky*; 6,097), “*freudig*” (*cheerful*; 558), “*vergnügt*” (*happy*; 483), “*spaßig*” (*funny*; 129), “*mutig*” (*brave*; 1,328), “*stolz*” (*proud*; 4,315), “*verliebt*” (*loving*; 2,300), “*fröhlich*” (*merry*; 1,862), and “*froh*” (*joyful*; 5,424), with a mean word length of Ø = 6.5 letters (range 4–9 letters) and an average frequency of Ø = 2,502. We used the following negative valence prime and target words: “*furchtsam*” (*fearful*; 59), “*ängstlich*” (*anxious*; 775), “*bekümmert*” (*worried*; 154), “*traurig*” (*sad*; 2,646), “*zornig*” (*furious*; 447), “*hasserfüllt*^2^” (*full of hate*; 339), “*wütend*” (*enraged*; 1,549), “*frustriert*” (*frustrated*; 899), “*beschämt*” (*ashamed*; 202), and “*schuldig*” (guilty; 4,753), with a mean word length of Ø = 8.3 letters (range 6–11 letters) and an average frequency of Ø = 1,182. The spatial primes that we used as up-words were: “*oben*” (*on top*; 21,453), “*darüber*” (*above*; 45,943), “*hinauf*” (2,214), “*aufwärts*” (1,652), “*empor*” (*upward*; 714), “*hoch*” (*high*; 27,559), “*gehoben*” (1,555), “*erhöht*” (*elevated*; 14,891), “*aufsteigend*” (143), “*steigend*” (*rising*; 750), with a mean word length of Ø = 6.6 letters (range 4–11 letters) and an average frequency of Ø = 11,687. Finally, the spatial primes that we used as down-words were: “*unten*” (*down*; 11,971), “*darunter*” (*below*; 22,589), “*hinab*” (1,024), “*abwärts*” (767), “*herab*” (*downward*; 1,624), “*niedrig*” (*low*; 3,529), “*gesenkt*” (*lowered*; 5,027), “*abfallend*” (see text footnote 2; 143), “*sinkend*” (“*declining*”; 60), and “*tief*” (*deep*; 10,331), with a mean word length of Ø = 6.3 letters (range 4–9 letters) and an average frequency of Ø = 5,707. These words were selected because of their relatively similar distributions in text corpora, a relatively similar length, and on the basis of their easy and equal discriminability of category-membership (which was empirically tested during pre-testing).

Each of the 20 valence words was presented as a target equally often. For the creation of the within-category priming condition, each target was randomly combined with each of the nine remaining valence words. For the across-category priming condition, each of the valence targets was randomly combined with each of the 10 spatial words as a prime. Across trials, the different prime words were equally likely and the resulting prime-target pairs were equally likely to be congruent or incongruent. In a trial, prime and target were never identical even in congruent conditions. This was done to rule out repetition priming (Forster, 1998).

**Figure 1:**
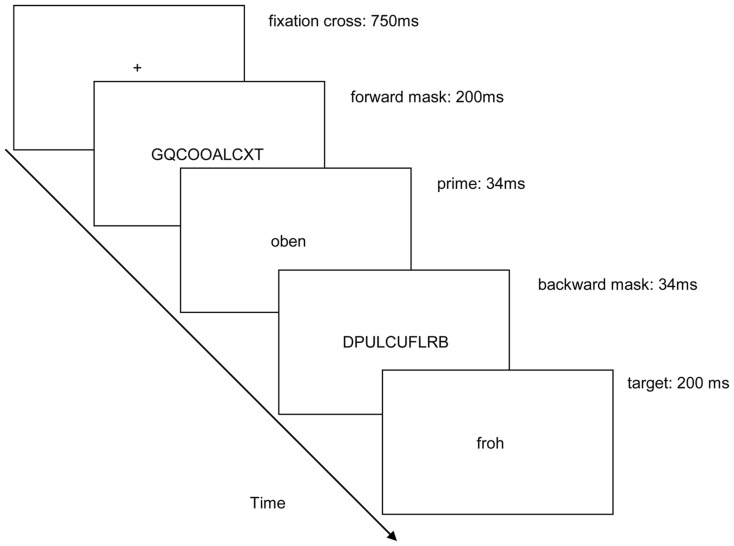
**Depicted is an example of a congruent trial where a spatial up-prime, here: “*oben*” (*on top*), preceded a positive valence target, here: “*froh*” (*joyful*)**. The arrow depicts the direction of time. In the target-response blocks, participants had to categorize the targets. Only in the blocked prime visibility task at the end of each half of the experiment, the participants also had to additionally judge prime-target congruence. Stimuli are not drawn to scale.

All stimuli were presented in black (<1 cd/m^2^) on a gray background (24 cd/m^2^). Each trial started with the presentation of a fixation cross centered on the screen for 750 ms (see Figure 1). In masked trials, a forward mask was shown next for 200 ms. It consisted of 10 randomly drawn uppercase letters. The prime word was shown for 34 ms immediately after the forward mask or after the blank screen in the case of visible trials. The prime was depicted in lowercase letters. In masked trials, the prime preceded a backward mask. The backward mask also consisted of 10 randomly drawn capital letters that were shown for 34 ms. In visible trials, both forward and backward masks were omitted and the masking screens were replaced by blank screens. Next, the target word was shown for 200 ms. In masked trials, all words and the masks were shown centered on the screen directly one after the other. Timing of all stimuli was adapted from prior studies that exhibited little prime visibility in masked and good prime visibility in unmasked conditions (Kiefer and Brendel, 2006; Ansorge et al., 2010, 2011).

The experiment consisted of two blocked conditions, one block with masked primes, and a second block with unmasked primes. The order of the blocks was either masked block before unmasked block, or vice versa, with different block orders balanced across participants. Each block lasted about 30 min. In each trial of both the masked and the unmasked conditions, participants had two tasks, first a blocked target-response task and subsequently a blocked prime-discrimination task. During the target-response task, participants discriminated the meaning of the target word. Half of the participants pressed the right key for positive target words and the left key for negative target words. The other half of the participants pressed the left key for positive targets and the right key for negative targets. The second task was a prime visibility task. This task required that one key (say the right key) be pressed in trials, in which the prime was congruent to the target and the other key (say the left key) if the prime was incongruent to the target. The levels of the variable prime-target congruence that had to be judged were carefully explained to the participants with relevant examples in the instructions. This task was conducted in every trial, directly after the target-discrimination task, so that conditions in this task were exactly the same as in the target-response task. This task of discriminating between congruent and incongruent trials has two advantages as compared to a task of discriminating the prime’s meaning (e.g., its valence) alone. First, the task of discriminating congruent from incongruent trials requires processing of prime *and* target. It thus necessitates processing of the targets not only in the target-discrimination task but also in the prime visibility test. Because we are interested in understanding whether prime visibility in the target-discrimination task might account for any priming effect in these conditions, the congruence-incongruence discrimination task is thus more apt to answer the research question that we asked. Second and related, the congruence effect in the target-discrimination task can only be explained on the basis of supraliminal word processing if the critical characteristics of the corresponding conditions that created the priming effect can be correctly discriminated by the participants. This critical characteristic that is decisive for the priming effect – that is, whether a quick or a slow response can be given, is whether a trial is congruent or whether it is incongruent. Therefore, a fitting prime visibility test needs to assess the participants’ awareness of this critical difference rather than the participants’ awareness of a difference between the primes that is only related to this critical difference, such as the exact meaning of the prime word alone. In the prime visibility task, mappings of judgments to alternative (left and right) key presses were also fixed and balanced across participants.

After each incorrect response to the target and if the target-discrimination RT exceeded 1,250 ms, participants received feedback about their error or their too slow responses. Feedback took 750 ms. Thus, keeping a high accuracy and a fast response was mildly rewarded (i.e., saved 750 ms per trial). No feedback was given concerning the prime visibility task.

Each block consisted of 240 trials. In total (across blocks), this involved 60 trials of each combination of the 2 prime types (spatial primes; valence primes) × 2 prime-target congruence relations (congruent; incongruent). Prior to the first block, participants were carefully instructed about the target-response task and the prime-discrimination task. Also, prior to both blocks, the participants practiced the task for a minimum of 20 trials but they could also practice for another 20 trials if they wanted to practice more. During these practice phases, the procedure was explained verbatim in addition to the preceding written instructions, and in more detail if necessary (i.e., if there were questions).

### Results

Of all correct responses, 3.8% were excluded because these RTs deviated by more than 2 SDs from a respective condition’s and individual participant’s mean RT (with SD and mean RT computed separately for each of the conditions and individuals).

An ANOVA of the medians of the correct responses, with the within-participant variables congruence (congruent; incongruent), prime type (valence; spatial), visibility (masked; unmasked), and quintile of RT distribution (first to fifth quintile) led to the following results. Here and in the subsequent analyses, results were adjusted by Greenhouse–Geisser coefficients and the ε values are reported, if Mauchly tests indicated a deviation from sphericity.

A significant main effect of congruence, *F*(1, 39) = 83.81, *p* < 0.01, partial η^2^ = 0.68, was found, reflecting faster RTs in congruent (652 ms) than incongruent (669 ms) conditions. There was also a significant main effect of prime type, *F*(1, 39) = 14.35, *p* < 0.01, partial η^2^ = 0.27, indicating that responses after spatial primes were slightly faster (RT = 656 ms) than with valence primes (RT = 664 ms). Critically, we also found significant interactions between congruence and prime type, *F*(1, 39) = 13.93, *p* < 0.01, partial η^2^ = 0.26, and between congruence and prime visibility, *F*(1, 39) = 10.15, *p* < 0.01, partial η^2^ = 0.21.

Splitting up the data during follow-up ANOVAs, we confirmed a congruence effect with both types of primes, a strong across-category priming effect of the spatial primes, *F*(1, 39) = 81.07, *p* < 0.01, partial η^2^ = 0.68 (congruent RT = 644 ms; incongruent RT = 669 ms), and a smaller within-category priming effect of the valence primes, *F*(1, 39) = 7.83, *p* < 0.01, partial η^2^ = 0.17 (congruent RT = 660 ms; incongruent RT = 668 ms). Follow-up ANOVAs split up for visible and masked primes confirmed a stronger congruence effect with visible primes, *F*(1, 39) = 76.10, *p* < 0.01, partial η^2^ = 0.66 (congruent RT = 650 ms; incongruent RT = 673 ms), than with masked primes, *F*(1, 39) = 16.63, *p* < 0.01, partial η^2^ = 0.30 (congruent RT = 653 ms; incongruent RT = 664 ms).

**Figure 2:**
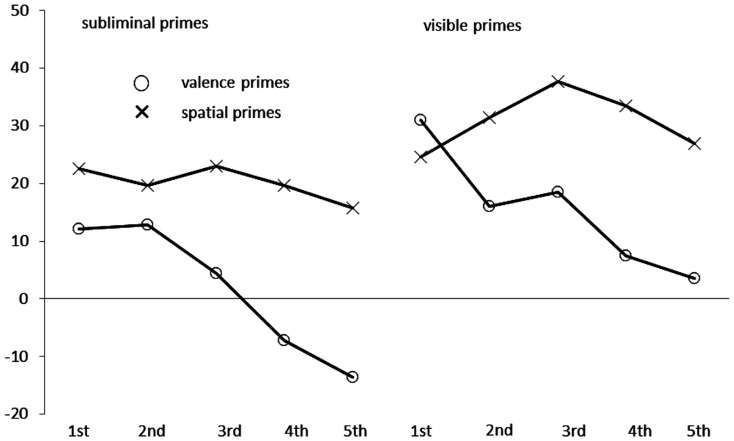
**Mean congruence effects in milliseconds, calculated as mean correct Reaction Times (RTs in ms) of incongruent conditions minus mean correct RTs of congruent conditions in Experiment 1, as a function of prime type (circles: valence primes; crosses: spatial primes), prime visibility (left side: subliminal primes; right side: visible primes) and quintile (first to fifth) of the RT distribution on the *x* axis**.

There was also a trivial main effect of the variable quintile, *F*(4, 156) = 270.59, *p* < 0.01, partial η^2^ = 0.87 (ε = 0.26), and the variable quintile also interacted significantly with congruence, *F*(4, 156) = 6.97, *p* < 0.01, partial η^2^ = 0.15 (ε = 0.51), with prime type, *F*(4, 156) = 5.80, *p* < 0.01, partial η^2^ = 0.13 (ε = 0.45), and in a marginally significant three-way interaction with congruence *and* prime type, *F*(4, 156) = 3.02, *p* = 0.07, partial η^2^ = 0.07 (ε = 0.38). As it can be seen in Figure 2, the within-category congruence effect (incongruent RT – congruent RT) of the valence primes (depicted as circles) decreased across RTs. It was significant only in the faster RTs [first quintile: 22 ms, *t*(39) = 5.96, *p* < 0.01, second quintile: 15 ms, *t*(39) = 4.43, *p* < 0.01, third quintile: 12 ms, *t*(39) = 3.61, *p* < 0.01] but it was absent among the slower response (fourth quintile: 0 ms, fifth quintile: −5 ms, both *t*s < 1.00). By contrast, the across-category congruence effect of the spatial primes (depicted as crosses in Figure 2) was fairly stable across RT. Across the RT distribution the congruence effect varied slightly in size between 20 and 30 ms (all *t*s > 2.70, all *p*s < 0.01). Together, these results are perfectly in line with the assumption that response-activation was responsible for (within-category) valence priming effects but categorization lay aground of spatial (across-category) priming because the valence primes were also response-relevant but the spatial primes were not.

An ANOVA of the error rates (ERs) with the variables congruence, prime type, and visibility confirmed the picture. The slower median correct reactions in incongruent than congruent conditions were accompanied by a lower mean accuracy in incongruent (ER = 6.5%) than congruent (ER = 5.3%) conditions. This was reflected in a significant main effect of congruence, *F*(1, 39) = 7.75, *p* < 0.01, partial η^2^ = 0.17. There was also a significant main effect of prime type *F*(1, 39) = 17.77, *p* < 0.01, partial η^2^ = 0.31 – with higher ERs for valence (6.6%) than spatial primes (5.2%), and a significant interaction between congruence and prime type, *F*(1, 39) = 20.62, *p* < 0.01, partial η^2^ = 0.35. Follow-up ANOVAs split up for the type of prime confirmed the existence of a significant main effect of congruence (3.4%) for spatial primes, *F*(1, 39) = 34.11, *p* < 0.01, partial η^2^ = 0.47, but not for valence primes (−0.7%, *F* < 1.00).

#### Supplementary information concerning polarity-correspondence effects

We also made sure that the within-category congruence effect did not merely reflect a polarity-correspondence effect (Lakens, 2012)^3^. According to this interpretation, as compared to the so-called “minus poles” (or “−poles”) of a meaning dimension (e.g., negative concepts in the valence dimension and down concepts in the spatial dimension), the so-called “plus poles” (or “+poles”) of meaning dimensions (e.g., positive concepts and up concepts) are processed faster and additionally facilitate +pole responses. The polarity-correspondence hypothesis would thus predict that prime-target combinations of concepts with similar polarities (e.g., +/+ prime-target combinations) are facilitated as compared to combinations with dissimilar polarities (e.g., −/+ prime-target combinations). Although this is true of the −pole concepts, too, the slower −pole processing and responses should lead to reduced congruence effects, with a congruent but very slow −/− prime-target combination being not so different of an incongruent but slightly facilitated +/− prime-target combination. As can be seen in Table 1 though, there was the expected facilitation for +targets but no strong difference between the congruence effects of +targets versus −targets.

**Table 1:** **Reaction times (in ms) as a function of prime-target combination in Experiment 1 and 2**.

	Experiment 1 (ms)	Experiment 2 (ms)
+/+ Prime-target	634	617
−/− Prime-target	655	623
−/+ Prime-target	645	643
+/− Prime-target	679	649

#### Prime visibility tests

To test whether participants consciously identified the unmasked primes but failed to see the masked primes, we computed *d*′, a sensitive index of stimulus visibility (Reingold and Merikle, 1988). Individual *d*′ was computed separately for masked and unmasked primes and for spatial primes and valence primes. For our measure of *d*′ congruent trials counted as signals and incongruent trials as noise. Accordingly correct (i.e., “congruent”) judgments in congruent trials figured as hits, and incorrect (i.e., “congruent”) judgments in incongruent trials as false alarms (FAs).

The participants were not able to discriminate the masked prime-target relations with better than chance accuracy. For the masked spatial primes, *d*′ was −0.10, *t*(39) = 1.08, *p* = 0.29, and for the masked valence primes it was 0.19, *t*(39) = 1.70, *p* = 0.10. In addition, the correlation between individual *d*′ values and individual Cohen’s *D* indices of congruence effects [calculated as (incongruent RT – congruent RT)/SD (pooled over congruent and incongruent) RT] indicated that there was no significant influence of residual prime visibility on the RT congruence effect for masked spatial primes, *r*(40) = 0.003, *p* = 0.99, and for masked valence primes, *r*(40) = 0.03, *p* = 0.86. The unmasked prime-target relations were successfully discriminated for the spatial primes, with *d*′ = 2.34, *t*(39) = 14.77, *p* < 0.01, and the valence primes with *d*′ = 2.82, *t*(39) = 15.26, *p* < 0.01.

### Discussion

In line with a quick influence of sense-related word meaning, an across-category space-valence congruence effect was found even with masked spatial primes^4^. This across-category congruence effect was created by subliminal words because the masked primes could not be seen by the participants. The effect is in line with the predictions of the embodied cognition view that assumes that a swift and (conditionally) automatic extraction of sensory meaning from a word could occur so fast as to influence emotions and evaluations (Niedenthal, 2007). The effect is also in line with the observed fast and automatic extraction of non-evaluative meaning before stimulus evaluation (Nummenmaa et al., 2010). In line with these observations, the participants’ processing of the spatial primes was so fast and efficient that an awareness of the primes was not a necessary precondition of the across-category congruence effect (Lamme, 2003).

The present experiment’s across-category priming effect of spatial words on valence discriminations might appear surprising in light of the finding of Meier and Robinson (2004) that discriminating between the spatially upper or lower position of a string of crosses on the computer screen had no influence on the discrimination of the valence of a subsequent word in the center of the screen: whether the discriminated position was congruent to a word’s valence or not had no systematic influence on valence discrimination in Meier and Robinson’s Experiment 3. However, Meier and Robinson asked their participants to respond first to the cross positions and only then to the valence targets whereas we asked our participants to first quickly respond to the valence targets. The use of the prime and the target in different tasks can be critical for the across-category congruence effect. For example, according to an explanation developed by Gozli et al. (2012), sorting prime and target into different tasks is one precondition that can lead to a reverted word-location congruence effect with short SOAs but not with long SOAs. Assuming that different RTs to the spatial primes in Experiment 3 of Meier and Robinson led to a mixture of short and long prime-target intervals, it could thus be that their lacking congruence effect reflected a mixture of straight and reverted congruence effect that averaged to zero. Whereas Meier and Robinson thus clearly sorted the cross positions and valence targets into separate tasks and created different prime-target intervals, we used a single task of discriminating a word’s valence and used a fix prime-target interval. Thus conditions for a straight across-category congruence effect were better in the current experiment than in Experiment 3 of Meier and Robinson.

Against our findings in Experiment 1, one might want to argue that the spatial primes were just implied by the set of task-relevant affective target words. According to this argument, the task of the participants to discriminate valenced emotional words, such as “sad” and “happy,” would have led the participants to also judge spatial words, such as “above” and “below,” by their respective connotative valence. In general agreement with this hypothesis, participants judge words, such as “up” as more positive, and words such as “down” as more negative (e.g., Eder and Rothermund, 2008). Although this alternative explanation is a theoretical possibility it would be difficult to reconcile this alternative explanation with the obvious differences between the congruence effects of the emotional primes and the spatial primes in the current study. Whereas the emotional primes led to a congruence effect that decreased across the RT distribution, this was not the case for the spatial primes. Therefore, the across-category congruence effect of the spatial primes was probably due to the priming of the target’s categorization. This was reflected in the development of the across-category congruence effect over time. The RT distribution reflected a fairly stable congruence effect of the spatial primes. According to Kinoshita and Hunt (2008) this would be typical of a category priming effect. In addition, the spatial words were also response-irrelevant in the first place because these words were not used as response-relevant targets anyway.

We also observed a weaker within-category congruence effect of the valence primes. This within-category priming effect probably reflected response-activation. To note, the valence words were used as primes and targets. Therefore, the valence primes were response-relevant. In line with this interpretation, the congruence effect of the valence primes decreased over RTs. It was stronger among the faster than the slower responses. This is typical of the response-activation effects of masked primes (Kinoshita and Hunt, 2008; Ansorge et al., 2010).

Finally, a significantly stronger congruence effect was found for visible than masked primes. The stronger influence of the visible primes probably reflected that their perceptual traces lingered longer and opportunities for attentional dwelling were thus higher with visible primes than masked primes. In line with this interpretation, when Santiago et al. (2012) directed their participants’ attention to the task-irrelevant valence meaning of words, they found a significant space-valence congruence effect that was absent when attention was not directed toward word valence.

## Experiment 2

Experiment 2 was our second test of the across-category priming effect. Some evidence suggests that the semantic content from images is extracted before an image can be evaluated (Nummenmaa et al., 2010). According to this line of thinking, the quick awareness-independent across-category congruence effect could be abolished when the roles of valence words and spatial words as primes and targets are reversed (as compared to Experiment 1).

We therefore reversed the roles of valence words and spatial words as across-category primes and targets. In contrast to Experiment 1, spatial words were now used as visible targets. The participants had to discriminate between up targets and down targets. As in Experiment 1, valence words as well as spatial words were used as primes. In this way, we were able to test whether masked valence primes created an automatic across-category space-valence congruence effect as would be predicted by a quick and automatic valence assessment of the words.

### Method

#### Participants

Four participants had to be excluded based on their above-chance discrimination of the masked primes^1^, and one because of very low performance even in the unmasked condition. The remaining 39 participants (29 female, *M*_age_ = 26.3 years, age range: 19–42 years) were analyzed.

#### Apparatus, stimuli, and procedure

These were the same as in Experiment 1, except for the changed targets and instructions. In Experiment 2, the participants were presented with space targets and they had to discriminate between up and down targets in the target-response task. Half of the participants responded to up targets by a right-hand key press and to down targets by a left-hand key press. The other half of the participants got the opposite mapping.

### Results

#### Target-response task

**Figure 3:**
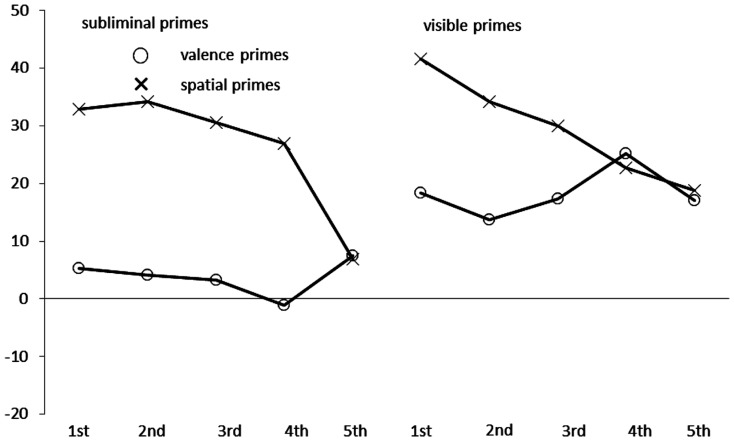
**Mean congruence effects in milliseconds, calculated as mean correct Reaction Times (RTs in ms) of incongruent conditions minus mean correct RTs of congruent conditions in Experiment 2, as a function of prime type (circles: valence primes; crosses: spatial primes), prime visibility (left side: subliminal primes; right side: visible primes), and quintile (first to fifth) of the RT distribution on the *x* axis**.

See also Figure 3. Of all correct responses, 4.0% were discarded by the same criterion as was used in Experiment 1. We ran an ANOVA of the correct RTs with the within-participant variables congruence, prime type, prime visibility, and quintiles. This ANOVA confirmed the significant main effects of congruence, *F*(1, 38) = 90.87, *p* < 0.01, partial η^2^ = 0.71, and an almost significant effect of prime type, *F*(1, 38) = 3.79, *p* = 0.06, partial η^2^ = 0.09, as well as the important interactions between (1) congruence and prime type, *F*(1, 38) = 23.21, *p* < 0.01, partial η^2^ = 0.38, (2) congruence and prime visibility, *F*(1, 38) = 5.34, *p* < 0.05, partial η^2^ = 0.12, and (3) congruence, prime type, and prime visibility, *F*(1, 38) = 4.62, *p* < 0.05, partial η^2^ = 0.11. Splitting up the data for follow-up analyses for different combinations of the steps of the variables congruence, visibility, and prime type (while collapsing across quintiles), we found significant congruence effects (incongruent RT – congruent RT) only for visible primes of either type, across-category valence primes [18 ms, *t*(38) = 6.25, *p* < 0.01] and within-category space primes [30 ms, *t*(38) = 6.63, *p* < 0.01], as well as for masked within-category space primes [26 ms, *t*(38) = 7.72, *p* < 0.01]. However, there was no significant congruence effect with the masked across-category valence primes [4 ms, *t*(38) = 1.17, *p* = 0.25].

Crucially, and in line with different origins of the congruence effects in the different prime type conditions, the ANOVA also revealed significant two-way interactions between quintile and congruence, *F*(4, 152) = 5.39, *p* < 0.01, partial η^2^ = 0.12, and between quintile and prime type, *F*(4, 152) = 6.26, *p* < 0.01, partial η^2^ = 0.14, as well as a significant three-way interaction between quintile, congruence, and prime type, *F*(4, 152) = 6.61, *p* < 0.01, partial η^2^ = 0.15. Figure 3 depicts these results. As it can be seen by looking at the cross symbols (depicting the spatial primes), in line with a motor priming effect of the spatial primes their congruence effect now decreased over RTs. In the spatial priming conditions, congruence effects were 37, 34, 30, 25, and 12 ms, all *t*s(38) > 2.20, all *p*s < 0.05, from the first to the fifth quintile, respectively. By contrast, looking at the circular symbols (depicting the valence primes), especially in the visible conditions, in line with a category priming effect, congruence effects of the visible valence primes were relatively similar over RTs. From the first to the fifth quintile the across-category valence priming effect fluctuated between 9 and 12 ms, all *t*s(38) > 2.20, all *p*s < 0.05.

Figure 3 also indicates that a four-way interaction between congruence, prime type, visibility, and quintile should have obtained – basically because the absence of the congruence effect with masked valence primes only – but this interaction fell short of significance, *F*(4, 152) = 2.10, *p* = 0.08, partial η^2^ = 0.05.

In addition we observed an interaction between quintile and prime visibility, *F*(4, 152) = 2.89, *p* = 0.07, reflecting a shallower slope (i.e., less variance) of the RT distribution in masked than in visible conditions, as well as a trivial main effect of quintile, *F*(4, 152) = 853.30, *p* < 0.01, partial η^2^ = 0.96.

In an ANOVA of the mean ERs, with the variables congruence, prime type, and visibility the main effects of congruence, *F*(1, 38) = 6.18, *p* < 0.05, partial η^2^ = 0.14 (congruent ER = 3.6%; incongruent ER = 4.3%), and prime type, *F*(1, 38) = 9.58, *p* < 0.01, partial η^2^ = 0.20 (spatial prime: ER = 4.5%; valence prime: ER = 3.4%), were also significant. These effects made clear that the congruence effect was not due to a speed-accuracy trade-off, whereas the faster RTs to spatial primes (compared to valence primes) came at the expense of higher ERs (for spatial primes than for valence primes). In addition, the two-way interaction of prime type and congruence was significant, *F*(1, 38) = 9.55, *p* < 0.01, partial η^2^ = 0.20, and there was a significant three-way interaction, *F*(1, 38) = 4.45, *p* < 0.05, partial η^2^ = 0.11. Follow-up *t*-tests to compare congruent with incongruent ERs, conducted separately for the different combinations of prime types and prime visibility, revealed that the three-way interaction reflected the same tendencies that we observed in the RTs, standard congruence effects (with advantages in congruent relative to incongruent conditions) in all priming conditions (unmasked/spatial primes: 1.1%; masked/spatial primes: 2.3%; unmasked valence primes: 0.3%) but a reverse congruence effect for masked valence primes (−1.7%).

#### Supplementary information concerning polarity-correspondence effects

This time, we did not even find the expected facilitation of +pole targets as compared to −pole targets and no difference in the respective congruence effects of these targets alike. For the results see Table 1.

#### Prime visibility tests

The masked primes were invisible as demonstrated by the participants’ mean chance performance. Mean *d*′ was not significantly different from zero. It was 0.02, *t* < 1.00, with the masked spatial primes, and it was 0.10, *t*(38) = 1.19, *p* = 0.24, with the masked valence primes. In addition, the correlation between individual *d*′ values and individual Cohen’s *D* indices of congruence effects demonstrated that the residual visibility of masked primes did not significantly affect RT congruence effects of masked spatial primes, *r*(39) = −0.07, *p* = 0.67, and of masked valence primes, *r*(39) = 0.07, *p* = 0.67. The same participants were able to discriminate between the unmasked prime-target relations. Mean *d*′ was 2.70, *t*(38) = 18.82, *p* < 0.01, with the unmasked spatial primes, and it was 3.43, *t*(38) = 30.87, *p* < 0.01, with the unmasked valence primes.

### Discussion

In Experiment 2, we found no significant across-category congruence effect based on masked valence primes. This is in contrast to the predictions based on quick (conditionally) automatic affective processes influencing semantic analysis and it is also in contrast to Experiment 1 in which we found an across-category congruence effect of the masked spatial primes. Together, the results point to an asymmetry between the sensory and the affective processing of the word meanings. Also, in line with prior research, an across-category effect of the visible valence primes showed that if strategic processing (or other forms of awareness-dependent processing) was allowed, we replicated the standard across-category congruence effect with the valence primes, too. Evidently, only the awareness-independent aspect of the valence-based across-category priming was prevented. This result fits well with recent findings from vision sciences, where it was found that image content is partly available before its evaluation (Nummenmaa et al., 2010).

In addition, our RT distribution analysis was suggestive of a category-based congruence effect of the valence primes and of a response-activation effect of the spatial primes. The congruence effect of the spatial primes decreased with an increasing RT which is typical of a response-activation effect (Kinoshita and Hunt, 2008; Ansorge et al., 2010). By contrast, the congruence effect of the visible valence primes was approximately the same for the different quintiles of the RT distribution which is the finger print of a category congruence effect (Kinoshita and Hunt, 2008).

Despite this qualitative similarity of the result patterns in Experiments 1 and 2, there were also a few important differences. First, the congruence effect of the visible spatial primes in the present experiment did not decrease to zero with an increasing RT, whereas the congruence effect of the visible valence primes in Experiment 1 was completely eliminated among the slowest responses. Second and related, in the current experiment, the residual congruence effect of the visible spatial primes in the slowest responses was of about the same size as that of the visible valence primes. These differences might reflect more average semantic congruence between different spatial words than between different emotional words, and could reflect unique sources of meaning differences between the congruent emotional words. Even valence congruent emotions, such as sadness and anger (both negative) or pride and loving (both positive) vary drastically according to further word meaning dimensions, like arousal (Wundt, 1896; Russell, 1980; which would be low for sadness but high for anger). These differences might have counteracted the valence-based congruence effect but no such diminishing influence seems to have been present with the spatial words.

## Experiment 3

It is also possible that the across-category priming effect of the spatial primes in Experiment 1 reflected a type of intention-independent or truly stimulus-driven priming effect instead of a conditionally automatic across-category congruence effect. Experiment 3 was therefore an additional control experiment. The control experiment was conducted to experimentally rule out an interpretation of Experiment 1’s across-category congruence effect in terms of a strongly automatic, bottom-up priming effect. As explained above, we assumed that Experiment 1’s across-category congruence effect probably reflected that the prime’s sensory meaning affected the task-relevant categorical evaluation of the valence targets as negative versus positive. If this was the case, it should be possible to abolish the across-category congruence effect. Past research has shown that participants can flexibly change their prime analysis in accordance with the instructions and the changing target categorization requirements (Klinger et al., 2000; Eckstein and Perrig, 2007; Norris and Kinoshita, 2008). For example, Klinger et al. (2000) asked their participants to classify the same visible targets as either positive versus negative targets in one condition but as animate versus inanimate targets in a second condition. These authors found that only the prime word meaning that was currently relevant for classifying the targets created an awareness-independent congruence effect. For example, if the participants classified the target words on the basis of the target’s valence, a target-congruently evaluated prime facilitated responses as compared to a target-incongruently evaluated prime. By contrast to this, it did not matter whether both prime and target were of the same animate or inanimate category or whether one denoted an animate object and the other an inanimate object. This pattern of results was reversed when the participants had to classify the targets on the basis of the targets’ category-membership to the categories of animate versus inanimate objects. Now the valence-based congruence effect was eliminated but a congruence effect on the basis of the status of the primes as names for animate versus inanimate objects was found.

From this it follows that we should be able to abolish a conditionally automatic across-category congruence effect when we no longer require categorization of the positive versus negative targets as two different categories. Here, we achieved this by changing the instructions and asking the participants to categorize both kinds of valence targets, negative and positive words, as belonging into the same class of objects. To that end, we used both valence and space words as primes *and* targets and asked our participants to categorize the targets into emotional adjectives on the one hand and into spatial prepositions on the other. Under these conditions, both negative and positive words belong to the same category. Hence, a conditionally automatic priming effect of the different spatial primes (i.e., up- versus down-words) on the categorization into negative and positive target words should be abolished because the difference between the emotional valences would no longer be relevant for the task at hand (Klinger et al., 2000; Eckstein and Perrig, 2007).

### Method

#### Participants

For the new experiment, one participant with epilepsy was not tested, and another eight participants failed on the prime-discrimination criterion^1^ as in Experiments 1 and 2. The remaining thirty participants (25 female, *M*_age_ = 24.1 years, age range: 21–38 years) were analyzed.

#### Apparatus, stimuli, and procedure

These were the same as in Experiment 1, except for the following differences. First, in the target-response task, participants had to discriminate between valence targets and spatial targets. Thus, in contrast to Experiment 1, all words were used as targets and as primes. However, to prevent combinatorial explosion, we used only the across-category prime-target combinations of major interest. (During prime-discrimination, participants had to judge whether prime-target pairs were space-valence congruent or whether they were space-valence incongruent.)

#### Target-response task

**Figure 4:**
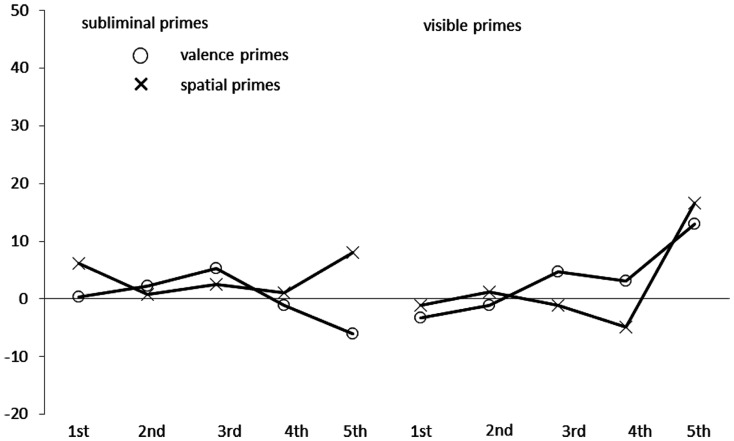
**Mean congruence effects in milliseconds, calculated as mean correct Reaction Times (RTs in ms) of incongruent conditions minus mean correct RTs of congruent conditions in Experiment 3, as a function of prime type (circles: valence primes; crosses: spatial primes), prime visibility (left side: subliminal primes; right side: visible primes), and quintile (first to fifth) of the RT distribution on the *x* axis**.

For the results, see also Figure 4. Of all responses, 4.2% were excluded by the same criterion as was used in Experiments 1 and 2.

In contrast to the preceding experiments, in an ANOVA with the variables congruence (space-valence congruent versus incongruent), prime type/target type (valence primes/spatial targets; spatial primes/valence targets), prime visibility (masked prime versus visible prime), and quintiles of the RT distribution (first to fifth quintile) there was no significant main effect of congruence, *F*(1, 29) = 1.08, *p* = 0.31, partial η^2^ = 0.04, and there was neither a significant interaction of congruence and prime type, *F* < 1.00, nor of congruence and prime visibility, *F* < 1.00. The same was true of the other interactions with the variable congruence, all *p*s > 0.15.

What we found were significant main effects of prime type, *F*(1, 29) = 12.65, *p* < 0.01, partial η^2^ = 0.30, of prime visibility, *F*(1, 29) = 4.75, *p* < 0.05, partial η^2^ = 0.14, and of quintile, *F*(4, 116) = 298.31, *p* < 0.01, partial η^2^ = 0.91, ε = 0.27. Responses were faster for spatial primes/valence targets (RT = 695 ms) than for valence primes/spatial targets (RT = 715 ms) and for visible primes (RT = 697 ms) than for masked priming conditions (RT = 714 ms). Also, RT increased over the RT distribution. In addition, there was a significant Prime Type/Target Type × Quintile interaction, *F*(4, 116) = 4.84, *p* < 0.05, partial η^2^ = 0.14, ε = 0.47, that was due to a steeper increase of the curves for space primes/valence targets than for valence primes/spatial targets. To put it differently, the faster RTs for spatial primes/valence targets that we observed in a main effect of prime type/target type were stemming from the fastest responses, whereas there were no large differences between the RTs in the different prime type conditions among the slower responses.

In the ANOVA of the ERs, we observed a significant interaction of congruence and prime type, *F*(1, 29) = 4.68, *p* < 0.05, partial η^2^ = 0.14. *Post hoc* *t*-tests showed that this was due to a non-significant “standard” congruence effect (0.7%), *t*(29) = 1.66, *p* = 0.1, for the spatial priming/valence target conditions and an almost significant “reverse” congruence effect (−1.1%) in the valence prime/spatial target conditions, *t*(29) = 1.89, *p* = 0.07. In addition, a significant interaction of prime type and visibility, *F*(1, 29) = 7.64, *p* < 0.01, partial η^2^ = 0.21, reflected that at least with the valence primes the faster RTs in unmasked than masked conditions (see RT ANOVA above) came at the expense of a higher ER in unmasked (7.2%) than masked (5.1%) conditions, *t*(29) = 2.50, *p* < 0.05, whereas with spatial primes no such difference was found [spatial primes: unmasked ER = 5.1%; masked ER = 5.4%, *t*(29) = 1.66, *p* = 0.11]. The main effects, all non-significant *F*s < 2.60, all *p*s > 0.19, all partial η^2^s < 0.09, and the remaining interactions, all non-significant *F*s < 1.10, all *p*s > 0.32, all partial η^2^s < 0.04, were not significant.

#### Prime visibility tests

Again, the participants were unable to discriminate the masked prime-target pairs. For the masked spatial primes, *d*′ amounted to 0.07, *t* < 1.00. For the masked valence primes, *d*′ was 0.13, *t*(29) = 1.51, *p* = 0.14. Participants were capable of discriminating the prime-target relations with the unmasked primes. For the unmasked spatial primes, *d*′ was 1.53, *t*(29) = 6.65, *p* < 0.01. For the unmasked valence primes, *d*′ amounted to 2.08, *t*(29) = 7.80, *p* < 0.01.

### Discussion

In Experiment 3, we wanted to rule out that the masked priming effect reflected a strongly automatic stimulus-driven effect. This was tested in this control experiment, in which all valence targets required one response and all spatial targets the alternative response. Under these conditions, if the masked priming effect was conditionally automatic, the spatial meaning of the primes should not have facilitated a categorization of the valence targets into negative versus positive words because valence discrimination was no longer required. As a consequence, the typical conditionally automatic categorization-congruence effect was expected to disappear because the categorization-congruence effect critically depends on an appropriate top-down categorization criterion on the side of the participants (Klinger et al., 2000; Eckstein and Perrig, 2007; Norris and Kinoshita, 2008).

An alternative prediction, however, was made for the control experiment if the congruence effect was due to stimulus-driven priming. If the spatial primes activated particular valence meaning in the preceding experiments in a stimulus-driven way, the congruence effect of the primes should have been found in the control conditions of Experiment 3, too, because the same primes and targets as in the preceding experiments were used.

At variance with this prediction, however, our results indicated that no congruence effect could be found in the present experiment. Thus, the data were much better in line with an origin of the across-category congruence effect in Experiments 1 and 2 via facilitation of the task-dependent target-category classification into positive versus negative words than via the stimulus-driven priming of one particular meaning.

Note also that the current experiment ruled out that the across-category priming effect in the supraliminal control conditions of Experiments 1 and 2 was an artifact of our procedure to ask the participants for a prime-target congruence judgment in the prime visibility test. One might want to argue that asking the participants to categorize primes and targets as congruent or incongruent in the across-category priming conditions created the supraliminal across-category priming effect (in the second blocks – that is after the first prime visibility test at the end of the first block) of the preceding Experiments 1 and 2 in the first place. If this would have been the case, the same across-category congruence effect should have been found in the supraliminal conditions of the present experiment because the same prime-target visibility judgment as in Experiments 1 and 2 was also required in the present Experiment 3. However, in contrast to this prediction, no across-category congruence effect could be found in the supraliminal conditions of the present experiment either. These results are better in line with an explanation of the across-category congruence effect in terms of the facilitating and/or interfering influence of spatial meaning during the participants’ discrimination between different positive and negative word valences.

## General Discussion

Since about 20 years, an increasing number of experiments firmly established the existence of a space-valence congruence effect (Lakoff and Johnson, 1980). The space-valence congruence effect reflects an advantage for the classification of and responses to combinations of associated (or congruent) spatial and affective meaning, such as the classification of positive words at elevated locations, as compared to less associated (or incongruent) meaning, such as negative words at elevated locations (Meier and Robinson, 2004). Such across-category congruence effects are potentially very informative with respect to the connection between cognition and emotion (Eder et al., 2007).

One particular question that haunts researchers in this domain is the sequence of events during semantic analysis of words in general and the meaning-connected evaluations in particular. On the one hand, evaluations of words and objects are very swift and automatic (Arnold, 1960) and they can occur before or outside of awareness (Naccache et al., 2005). On the other hand, some semantic information also seems to lay aground of subsequent evaluations and therefore some forms of non-evaluative meaning extraction might have to precede evaluations (Storbeck and Clore, 2007). In line with this view, non-evaluative semantic classifications are sometimes faster than evaluative classifications (Nummenmaa et al., 2010) and non-evaluative and evaluative semantic classifications can both occur independently of awareness, too (Kiefer, 2002).

To investigate these issues during lexical access to word meaning in general and the case of the space-valence association in particular, we used subliminal priming with words as primes and targets. Subliminal priming of words allows measuring of an awareness-independent quick and (conditionally) automatic congruence effect based on the degree of congruence or fit between subliminal priming word and to-be-classified target word. Here, this method was used to investigate one particular source of non-evaluative semantic impact on valence assignments that has been emphasized as important by the defenders of an embodied cognition view on emotions: the impact of sense-related (or sensory) non-evaluative meaning on emotions (Niedenthal, 2007). If the quick extraction of sensory non-evaluative meaning impacts on emotions, we expected an across-category priming effect of subliminal (masked) spatial prime word meaning on the classification of the valence of the visible target words. A corresponding awareness-independent across-category congruence effect was found in the present Experiment 1.

In addition, if evaluations also occur so quickly as to influence non-evaluative semantics used in sense-related classifications of words, we expected a similar congruence priming effect of subliminal valence words on the classification of the spatial elevation-meaning of visible target words. In contrast to this prediction, however, this across-category congruence effect was not found with subliminal valence word primes (Experiment 2). The across-category congruence effect of the valence prime words on the categorization of the spatial target words was only found with clearly visible supraliminal valence primes. The latter effect presumably reflected a strategic processing elicited by the prime words themselves and was therefore dependent on the use of clearly visible valence prime words (Forster, 1998). Such strategic processing of the visible prime words concerns the alteration of the prime processing that owes to their conscious recognition. For example, visible primes might have offered more opportunities for the participants’ attentional dwelling on their meaning than masked primes and the amount of attention indeed seems to be an important mediator for space-valence congruence effects (Santiago et al., 2012). Related, once the participants can see the primes, participants might want to learn whether the prime categories predict the target categories. If the participants adopt such a strategy, they will be willingly processing both the meaning of the prime words and that of the target words. As a consequence, the prime meaning can then influence processing of the target meaning even in valence-space across-category prime-target word pairings where this kind of influence would otherwise not be possible. Together, the significant across-category congruence effect of the supraliminal primes and the absent congruence effect of the subliminal primes in the conditions with valence primes and spatial targets also made clear that a qualitative difference existed between aware and unaware processing modes.

In addition, in Experiments 1 and 2, we also found motor-activation effects of the primes in the within-category priming conditions. Here, the prime words were from the same set as the target words so that the prime words had the power to elicit a target-associated response alternative (Klinger et al., 2000; Kunde et al., 2003). In these conditions, a quickly dissipating congruence effect was found, also for the subliminal valence words (see Experiment 1). The fact that this congruence effect decreased over RT was in line with its assumed origin on a response-activation level (Kinoshita and Hunt, 2008). Note that this kind of response-activation effect probably reflected the task-dependent motor meaning of the words. Therefore, these motor priming effects could not account for the across-category congruence effects in Experiments 1 and 2 in which the priming words were from a different category than the response-relevant target and were never used as response-relevant stimuli (Naccache and Dehaene, 2001; Klauer et al., 2007). In addition, no stimulus-driven automatic across-category priming effect could be found once the valence discrimination was no longer required (Experiment 3).

Moreover, two further observations suggested that the awareness-independent across-category congruence effect reflected an influence of the primes on the targets’ semantic categorization. First, the fact that the across-category congruence effect in Experiment 3 was absent is in line with its origin on the level of a category priming effect because subliminal semantic category priming effects are conditional on a fitting task set (Kunde et al., 2003; Eckstein and Perrig, 2007; Norris and Kinoshita, 2008). Second, in Experiments 1 and 2 the across-category congruence effect in the subliminal and supraliminal priming conditions were more or less of a similar strength across the RT distribution (Kinoshita and Hunt, 2008). Jointly, our data were thus in line with a swift and awareness-independent influence of semantic processes on evaluations in general (Storbeck and Clore, 2007; Nummenmaa et al., 2010), and of sense-related non-evaluative meaning on emotions in particular (Niedenthal, 2007).

This is not to say, however, that awareness-independent processing of non-evaluative meaning always has to precede evaluative processing. To note, we have studied the sequence of non-evaluative versus evaluative meaning extraction with regard to only one particular class of stimuli (words) and one particular type of non-evaluative meaning (spatial meaning). We can therefore not tell whether a similar sequence holds with other stimuli and alternative types of meaning. It is possible for example that specific valence stimuli, such as emotional facial expressions, are processed as quickly or even quicker than certain stimuli with a non-evaluative meaning. In line with this assumption, for example, participants are able to process subliminal facial expressions (Jolij and Lamme, 2005; Smith, 2011) even without a specific categorization task (Naccache et al., 2005). In fact, sensory or sensorimotor representations of affective facial expressions could be one valence-specific way of the embodiment of evaluative meaning (Niedenthal, 2007) – that is, the distinction of evaluative and non-evaluative meaning might not be feasible with regard to stimuli, such as human faces.

One further aspect that requires a brief discussion is our visibility measure. One might argue that with the current procedure of asking the participants to classify prime-target pairs after the preceding target responses, we could have underestimated the masked primes’ visibility and prime judgments might have been influenced by the fluency of the target responses (e.g., prime judgments might have occurred earlier in congruent than incongruent conditions, allowing for more forgetting of priming information in incongruent conditions). However, when exactly the same timing, sequence, size, luminance, and positioning of the primes and masks were used with different prime visibility tasks in a preceding study, the same results were found: prime visibility is also zero if the prime judgments are given in separate blocks of trials without preceding target responses, and if the prime judgment concerns the classification of the primes alone instead of the prime-target categorization (Ansorge et al., 2010). Thus, we are confident that the present finding of prime invisibility in the masked priming conditions is not just an artifact of the particular method that we have chosen.

As a final cautionary remark, however, an asymmetry of the masked priming effect (of space concepts on valence but not of valence on space) as we have found it might also be in line with some characteristics of language use, such as frequent metaphorical reference to abstract concepts (here: valence) by more concrete concepts (here: spatial meaning; Lakoff and Johnson, 1980; Santiago et al., 2012), whereas the asymmetry of masked priming effects might be at variance with some particular explanations of the space-valence congruence effect (Walsh, 2003; Meier and Robinson, 2004).

## Conclusion

The present study shows that subliminal spatial words affected classification of associated valence word meaning but that there was no corresponding influence of subliminal valence words on the classification of spatial word meaning. This was different with supraliminal words where the influences of space on valence and of valence on space were reciprocal. Jointly, these data suggest that spatial word meaning can precede access to valence word meaning to create space-valence associations in word understanding, and that space-valence meaning associations with aware and unaware words are owing to partly different processing strategies.

## Author Note

The authors declare shared first-authorship of Ulrich Ansorge, Faculty of Psychology, University of Vienna, Vienna, Austria, and Shah Khalid, Institute of Cognitive Science, University of Osnabrück, Osnabrück, Germany. Supported by Deutsche Forschungsgemeinschaft Grant An 393/5-1 to Ulrich Ansorge, Werner Klotz and Ingrid Scharlau, and by European Research Council’s ERC-2010-AdG #269716 – MULTISENSE to Peter König. Thanks to Daniela Egerer, Katharina Sehling, and Caroline Rott for help with the data collection.

## Conflict of Interest Statement

The authors declare that the research was conducted in the absence of any commercial or financial relationships that could be construed as a potential conflict of interest.

## Footnotes

^1^To ensure that the primes were not seen, individual *d*′ scores were computed as a participant’s difference between her/his *z*-transformed hit rate and the *z*-transformed FA rate during prime-discrimination. The *d*′ score becomes zero for chance performance and it can infinitely increase with an increasing number of correct judgments. Participants with an individual *d*′ score above the confidence interval, calculated according to equation (1) (Macmillan and Creelman, 2005) were excluded from the analysis because of their potential capability to see the masked prime:

(1) $$\Phi \left(p\right)={\left(2\pi \right)}^{-1/2}\exp\left[-0.5z{\left(p\right)}^{2}\right].$$

^2^Word counts of “*hasserfüllt*” included the word “*hass*” (“*hatred*”) and word counts of “*abfallend*” included the word “*fallend*” (“*falling*”).

^3^We are grateful to Daniël Lakens for pointing these predictions out to us.

^4^We were concerned that the congruence effect of the subliminal spatial primes could have reflected transfer of priming effects from preceding unmasked to masked blocks. Therefore, a complementary ANOVA was run with the additional between-participants variable block sequence (masked primes first vs. unmasked primes first), and the within-participant variables congruence, prime type, and visibility, as before. In addition to the significant results of the primary ANOVA, we observed a significant three-way interaction of sequence, prime type, and prime-target congruence, *F*(1, 38) = 4.19, *p* < 0.05, and partial η^2^ = 0.10. This three-way interaction reflected that a significant congruence effect (incongruent RT minus congruent RT) was found for all prime types [all significant congruence effects > 11 ms; all significant *t*s(19) > 2.40, all significant *p*s < 0.05) but for the affective primes when unmasked primes were presented in the first block [congruence effect = 8 ms; *t*(19) = 1.35, *p* = 0.19]. Thus, the across-category priming effect of the masked spatial primes was not due to transfer from preceding visible priming blocks only.
